# Optimizing the transition of care in pediatric kidney transplant recipients at a tertiary center: strategies to enhance autonomy and treatment adherence

**DOI:** 10.3389/fped.2026.1971206

**Published:** 2026-10-30

**Authors:** Maria Sangermano, Martina Donadello, Maria Rizzo, Elena Marinelli, Cristiana Ciato, Ester Valentini, Francesca Zennaro, Giorgio Pozziani, Germana Longo, Chiara Caletti, Marianna Alessi, Enrico Vidal, Elisa Benetti

**Affiliations:** 1 Department of Women's and Children's Health, Pediatric Nephrology Unit, Padua University Hospital, Padova, Italy; 2 Department of Medicine, Division of Nephrology, University of Verona, Verona, Italy; 3 Department of Medicine, Dialysis and Transplantation Unit, Padua University Hospital, Padova, Italy

**Keywords:** kidney transplantation, pediatric, readiness, transition, young adult

## Abstract

Transition from pediatric to adult care represents a vulnerable period for adolescent kidney transplant recipients. This study describes the implementation and preliminary evaluation of a structured multidisciplinary transition program at a tertiary-care university hospital. Transition readiness was assessed using an institutional transition-readiness questionnaire, completed by adolescents and parents, together with clinician-reported transition readiness assessments by pediatric and adult nephrologists. Among 51 screened kidney transplant recipients, 34 were eligible, and 10 adolescents (mean age 18.1 ± 1.4 years) completed the assessment. Overall readiness was high, with 74.4% of responses indicating full autonomy (score 1) and a mean score of 1.3 on a three-point scale. Readiness was highest in emotional regulation, lifestyle, and communication, whereas lower autonomy was observed in medical care management, particularly medication organization and healthcare navigation. Parents perceived adolescents as less autonomous than adolescents’ self-assessments, while pediatric nephrologists tended to provide more cautious evaluations than adult nephrologists. A novel aspect of this study was the integration of adolescent, parent, pediatric nephrologist, and adult nephrologist perspectives into a multidimensional assessment of transition readiness. Structured multidisciplinary transition programs may support individualized transition planning, promote patient autonomy, and ensure continuity of care.

## 1 Introduction

Over the past decades, major advances in medical and surgical management have improved the prognosis of children undergoing kidney transplantation. Today, the majority of pediatric kidney transplant recipients reach adulthood with a functioning graft, shifting the clinical focus from survival to ensuring long-term graft preservation. However, the transition from pediatric to adult healthcare services remains one of the most vulnerable periods in the trajectory of kidney transplant recipients. Adolescents and young adults experience the highest rates of graft failure among all age groups, with a well-recognized “high-risk window” between 17 and 24 years of age that coincides with the transfer to adult care (1).

The concept of transition, first defined by Blum and colleagues as “the purposeful, planned movement of adolescents and young adults with chronic conditions from child-centered to adult-oriented health care systems,” differs fundamentally from the simple act of transfer (2). When transition is reduced to a single administrative event, patients may experience discontinuity of care, uncertainty, and reduced adherence to treatment (1, 3, 4). Several studies have shown that abrupt or poorly coordinated transfers are associated with reduced adherence to therapy, an increased risk of acute rejection, and graft loss. Psychosocial factors, including the desire for independence, limited self-management skills, and inadequate communication between pediatric and adult care teams, may further exacerbate these risks (3).

In response to these challenges, several structured transition models have been developed, although their organization varies considerably across centers. Multidisciplinary transition programs involving pediatric and adult nephrologists, nurses, psychologists, and social workers have shown promising results in improving continuity of care and patient satisfaction (5). Nevertheless, there is still no consensus regarding the optimal structure or core components of transition programs. Recent consensus statements from the European Renal Association and the European Society for Paediatric Nephrology recommend structured, individualized transition pathways while acknowledging the limited evidence supporting specific models and interventions (6).

The present study aims to contribute to the growing body of evidence on transitional care by describing the development and preliminary evaluation of a structured transition program for adolescent kidney transplant recipients at a tertiary-care university hospital pediatric kidney transplant center.

## 2 Materials and methods

### 2.1 Study design and setting

This study was conducted in two consecutive phases. The first phase consisted of a nursing-led assessment of transition readiness using a structured questionnaire administered to adolescents with chronic kidney disease, including transplant recipients, and their parents. The assessment evaluated multiple domains of transition readiness, including disease knowledge, medication management, healthcare utilization, communication with healthcare professionals, lifestyle, emotional management, and social support.

Based on the findings of the initial assessment, a structured multidisciplinary transition program specifically designed for adolescent kidney transplant recipients was implemented in 2024. The program integrated medical, nursing, psychological, educational, and social interventions aimed at supporting the progressive development of self-management skills, promoting patient empowerment, and ensuring continuity of care between pediatric and adult nephrology services.

The present study combines the results of the initial readiness assessment with the preliminary evaluation of the structured transition program. The two phases involved partially overlapping but distinct study populations and were analyzed separately. By integrating these complementary phases, the study aimed to characterize transition readiness in adolescent kidney transplant recipients and to provide a comprehensive evaluation of the early implementation of a structured multidisciplinary transition program. The study was approved by the local Ethics Committee, and written informed consent was obtained from all participants and/or their legal guardians.

### 2.2 Transition program

The structured transition program developed for adolescent kidney transplant recipients in 2024 follows a stepwise model aimed at preparing patients and families for transfer to adult nephrology care. The process is organized into three phases:

1. Preparation (pre-transition): patients and families receive informational materials about the transition process, and the readiness questionnaires are administered to assess baseline knowledge and autonomy.
2. Evaluation (active transition): the multidisciplinary team (pediatric and adult nephrologists, nurses, and a clinical psychologist) reviews and discusses the questionnaire results to identify individual strengths and areas for improvement. Targeted educational interventions are then planned.
3. Completion (post-transition): the final pediatric nephrology visit is conducted jointly with the adult nephrologist, followed by the first appointment with the adult nephrology service, attended by the patient and family to ensure a smooth and supported handover.

The individual transition pathway begins with a dedicated visit during which the program is introduced to the patient and family, informed consent is obtained, and transition readiness is assessed using a structured questionnaire. During the pre-transition phase, educational and empowerment interventions are tailored to individual needs and focus on the progressive acquisition of disease- and transplant-related knowledge, self-management skills, and autonomy in navigating the healthcare system. The transition phase includes at least two joint visits involving the pediatric and adult nephrologists, one in the pediatric setting and one in the adult care setting. Nursing staff, psychologists, social workers, and other healthcare professionals are involved according to individual patient needs. At transfer, a kidney transplant-specific transition checklist/transition report is completed to provide the adult team with a structured summary of the patient's clinical history and current management (Supplementary Material–Appendix 1). Following transfer to adult care, a post-transition assessment is performed at 6 months to evaluate adherence and satisfaction with the transition process. The overall structure and components of the transition pathway are summarized in Table 1.

**Table 1:** Structure and components of the transition program.

Phase	Main activities	Professionals involved	Tools/resources
Pre-transition	Introduction to transition pathway; informed consent; readiness assessment; individualized education and empowerment	Pediatric nephrologist, nursing staff ±	Transition-readiness questionnaire
Transition	Preparation for transfer and progressive acquisition of autonomy	Pediatric and adult nephrologists; multidisciplinary team (psychologist/social worker/other professionals) as needed	Individualized educational interventions
Transfer	At least two joint visits: pediatric setting and adult setting; structured clinical handover	Pediatric and adult nephrologists	Kidney transplant transition checklist/transition report
Post-transition	Assessment after transfer	Adult team ± pediatric team	Follow-up assessment

### 2.3 Participants

In the first phase, the study population included adolescents and young adults who were approaching the transition to adult care. Patients were considered eligible if they were between 17 and 23 years of age, had a diagnosis of chronic kidney disease (CKD), whether or not they had undergone kidney transplantation, were under regular follow-up, and had been informed by the medical team about the possibility of transfer to adult nephrology services. Exclusion criteria included cognitive delay or neurodevelopmental disorders and insufficient knowledge of the Italian language.

The second phase of the project included kidney transplant recipients aged 16–20 years. Eligible patients were required to have a stable clinical condition for at least six months, a sufficient level of autonomy and motivation, and a supportive family environment. Exclusion criteria included severe neurocognitive impairment, unstable psychosocial conditions, terminal or pre-terminal disease, late referral to our Unit.

### 2.4 Assessment tool

Transition readiness was assessed using an institutional questionnaire developed within the transition pathway of Padua University Hospital and subsequently reviewed and adapted by the multidisciplinary transition team for use in pediatric nephrology, including adolescents with CKD and kidney transplantation. Item selection and adaptation were based on the clinical relevance of individual competencies to progressive healthcare autonomy and on the organization of the local healthcare system. The resulting 39-item questionnaire assesses disease and treatment knowledge, medication self-management, healthcare utilization and navigation, communication with healthcare professionals, lifestyle and daily activities, family and social support, and emotional well-being. (Supplementary Material–Appendix 2).

These areas are consistent with major transition-readiness domains described in established generic and kidney transplant-specific instruments, including the Transition Readiness Assessment Questionnaire (TRAQ) (7), the Readiness for Transition Questionnaire (RTQ) (8), and transplant-specific transition readiness checklists (9). Parallel adolescent and caregiver versions were used to explore both patient-reported readiness and caregiver perceptions (Supplementary Material–Appendix 3).

### 2.5 Assessment of clinician-reported transition readiness

Transition readiness was also assessed by pediatric and adult nephrologists using a 21-item clinician-reported transition-readiness questionnaire, evaluating disease knowledge, treatment adherence, healthcare navigation, and psychosocial autonomy (Supplementary Material–Appendix 4). Items were scored on a 3-point Likert scale, with higher scores indicating lower readiness (total score 21–63: 21–30 high, 31–45 intermediate, >45 low readiness). Pediatric nephrologists completed the questionnaire before transfer, whereas adult nephrologists completed it after at least 6 months of follow-up. Scores were analyzed descriptively and compared when both evaluations were available.

### 2.6 Clinical data and post-transition clinical outcomes

Demographic and clinical data, including age, sex, underlying diagnosis, time since transplantation, and graft function, were collected from the patients’ medical records. Clinical outcomes after transfer to adult care were retrospectively collected from medical records. Follow-up variables included graft function after transfer, episodes of biopsy-proven or clinically suspected rejection, major infectious complications, graft loss, and return to dialysis. Post-transition outcomes were analyzed descriptively and explored in relation to clinician-reported readiness scores.

### 2.7 Data analysis

Questionnaire data were compiled using Google Forms and analyzed with Microsoft Excel (Office 2016). All questionnaires included in the analysis were complete, with no missing item responses. Descriptive statistics were used to evaluate the results, calculating means and standard deviations for continuous variables, and absolute and relative frequencies for categorical variables. No inferential statistical tests were applied, as the analysis was intended to provide a descriptive overview of patients’ transition readiness profiles.

## 3 Results

### 3.1 Study population

In the first phase, 29 adolescents and young adults with CKD participated in the transition readiness assessment (15 males and 14 females; mean age 19.0 ± 2.2 years). All participants were Italian nationals. The mean age at diagnosis of kidney disease was 11.0 ± 5.6 years. Eighteen participants (62.1%) were kidney transplant recipients, while the remaining 11 (37.9%) were receiving conservative treatment or dialysis. Most parents had completed secondary education, and the mean parental ages were 49 years for mothers and 52 years for fathers.

In the second phase, 51 pediatric kidney transplant recipients were screened for inclusion in the structured transition program. After exclusion of patients with severe neurocognitive impairment or ongoing transfer to adult care, 34 patients met the eligibility criteria. At the time of this preliminary analysis, 10 patients had completed both the patient and parent questionnaires and were included in the readiness assessment (Figure 1). The cohort comprised 9 males and 1 female, with a mean age of 18.1 years (SD 1.4; range 16–20 years). Mean time since transplantation was 9.3 years (SD 5.1; range 2–16 years). The underlying causes of kidney failure were predominantly congenital anomalies of the kidney and urinary tract (CAKUT) (60%), followed by nephronophthisis, acute tubular necrosis, multiple organ failure, and unknown etiology (10% each).

**Figure 1:**
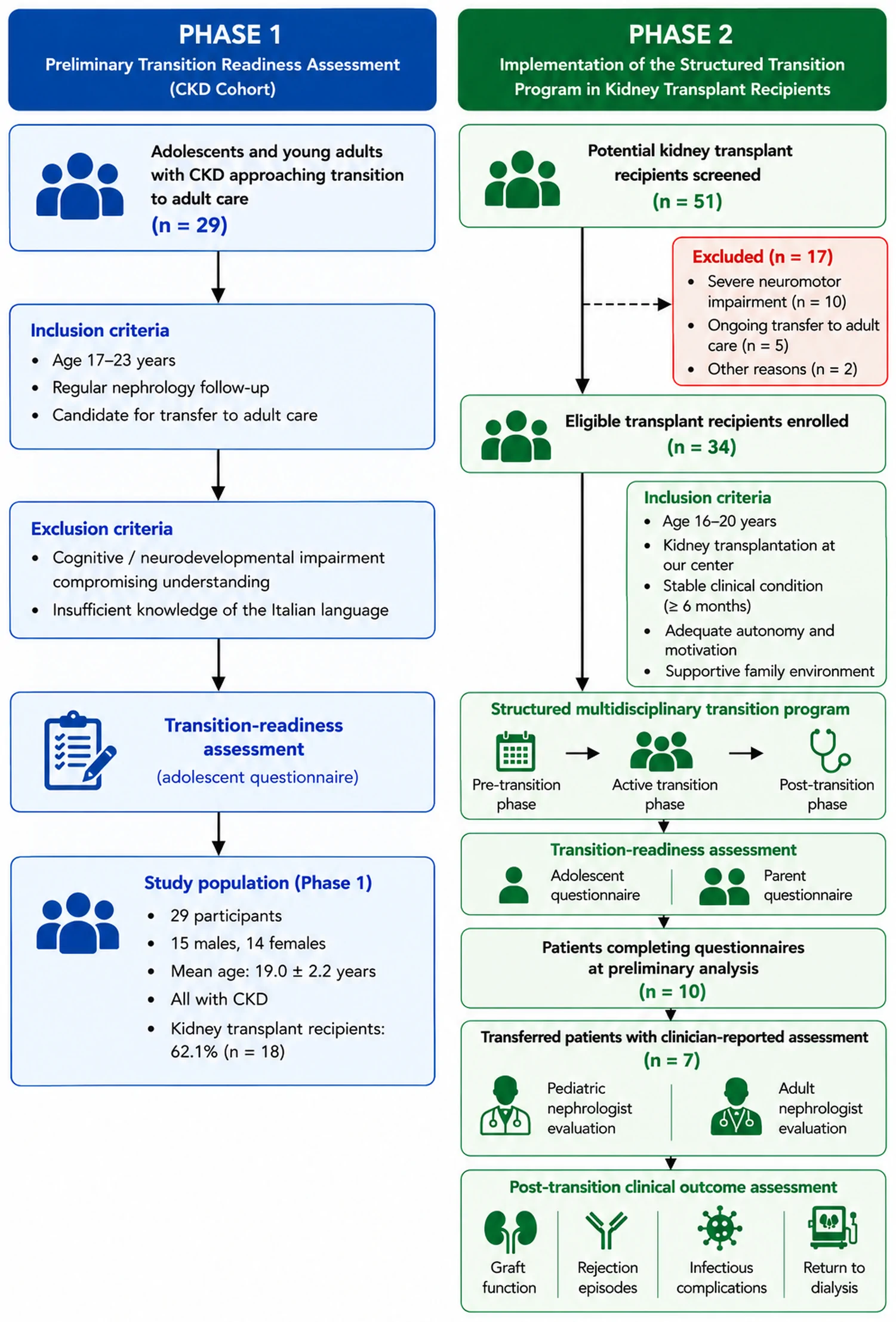
Study design and patient flow of the structured transition program. The figure illustrates the two phases of the study, including patient selection, transition readiness assessment, implementation of the structured transition program, and post-transition outcome evaluation.

### 3.2 Nursing-led transition readiness assessment

#### 3.2.1 Medication management

Overall, patients displayed partial autonomy in the management of pharmacological therapy. Adherence to treatment appeared generally adequate, whereas prescription renewal, proactive medication management, and recognition of adverse drug reactions represented areas requiring further support and education (Figure 2).

**Figure 2:**
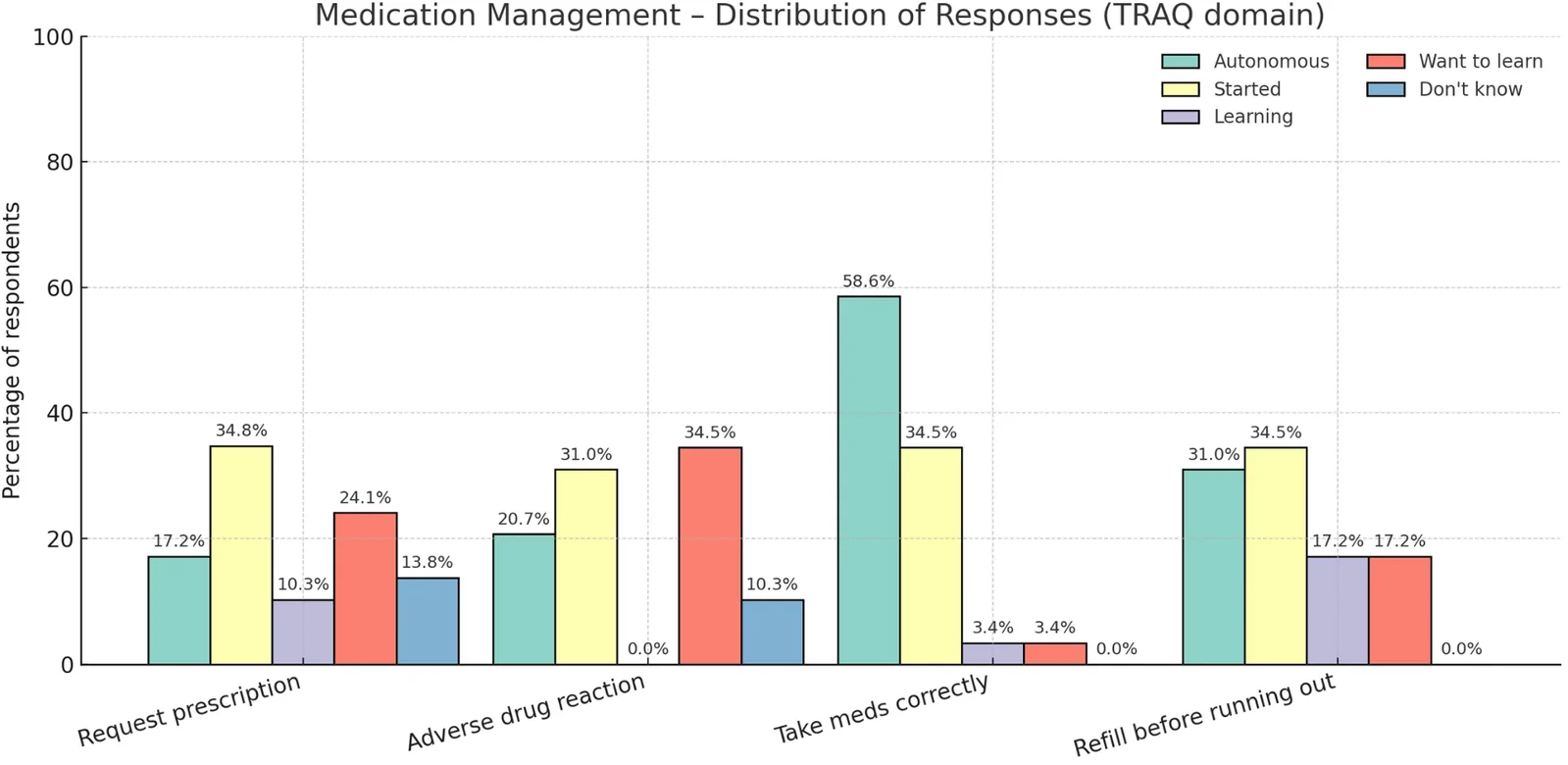
Distribution of responses in the “medication management” domain of the transition-readiness questionnaire. The figure shows the percentage of participants selecting each response category for the four items assessing medication management skills.

#### 3.2.2 Healthcare navigation

Transition readiness in healthcare organization and navigation skills was heterogeneous. Many participants reported limited confidence in independently scheduling appointments, organizing transportation for medical visits, and contacting healthcare professionals in the event of sudden health changes. Altogether, these results revealed a substantial gap in healthcare navigation skills (Figure 3).

**Figure 3:**
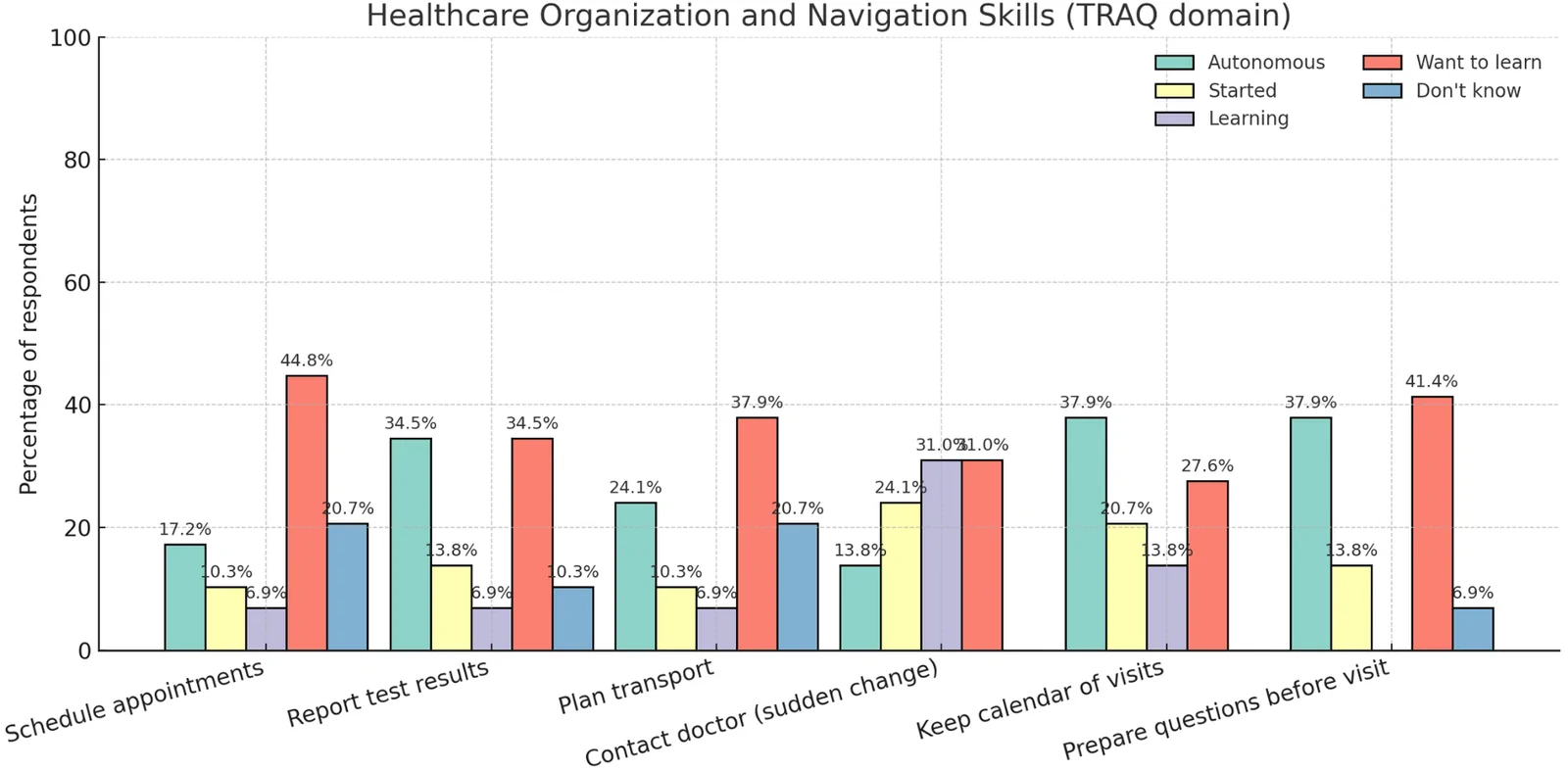
Distribution of responses in the “healthcare organization and navigation skills” domain of the transition-readiness questionnaire. Bars represent the percentage of participants selecting each response category for the six items assessing healthcare organization and navigation skills.

#### 3.2.3 Communication and daily living skills

Communication skills emerged as a major strength. Most participants reported confidence in discussing their health status directly with healthcare professionals and answering questions independently. Similarly, participants demonstrated a high degree of autonomy in daily activities, including meal preparation, household responsibilities, and financial management.

These findings indicated that adolescents are generally confident in verbal interactions with healthcare professionals and demonstrate good functional autonomy in everyday life, with the main needs concerning complex health management rather than daily self-care (Figure 4).

**Figure 4:**
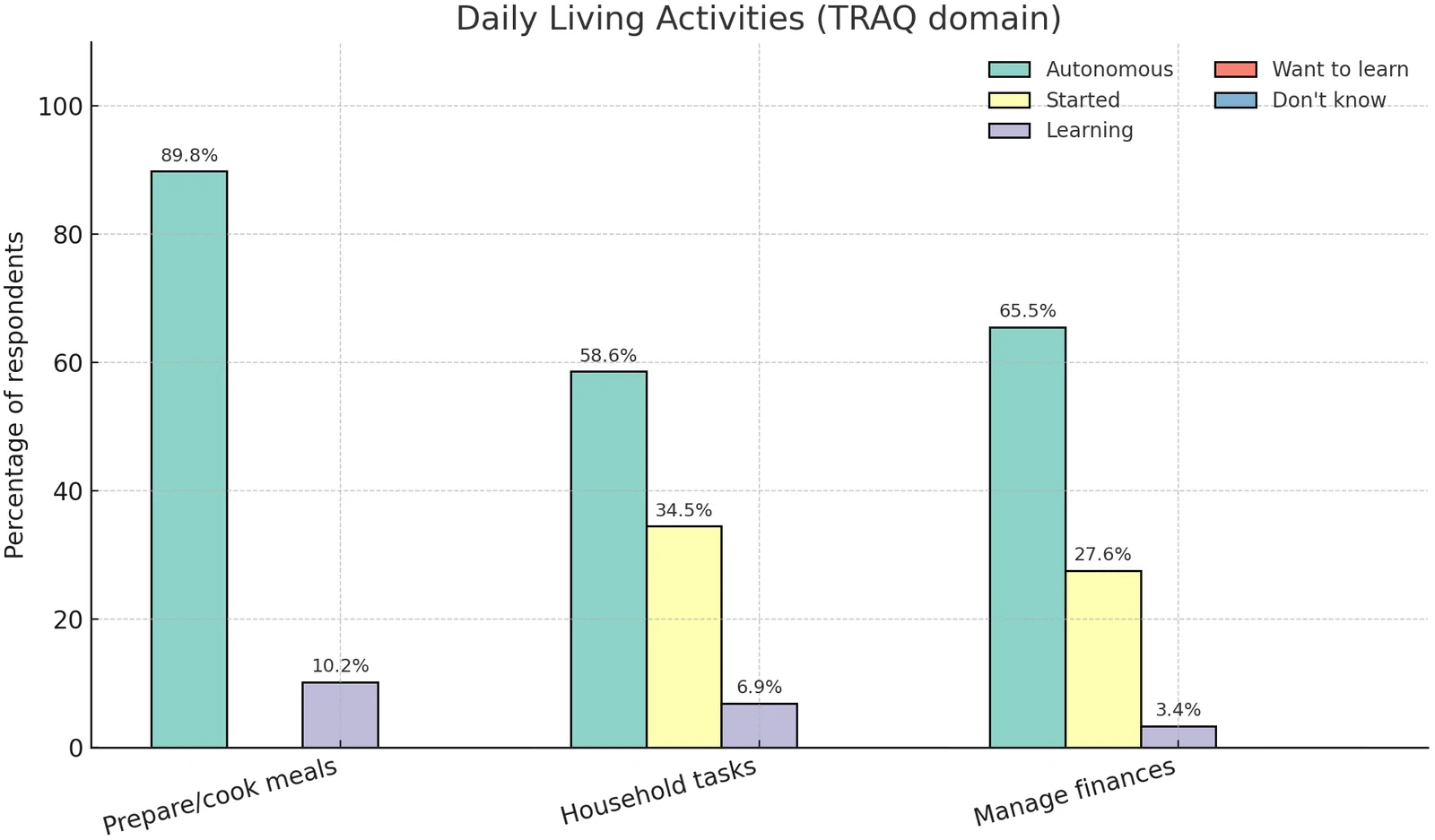
Distribution of responses in the “daily living activities” domain of the transition-readiness questionnaire. Bars represent the percentage of participants selecting each response category for the three items assessing daily living skills.

#### 3.2.4 Awareness of healthcare and financial support

Awareness of healthcare coverage, disability-related benefits, and financial support programs showed considerable variability among participants. While approximately half of respondents reported adequate knowledge of available services and entitlements, a substantial proportion expressed the need for additional information. Overall, these findings highlighted a heterogeneous level of awareness concerning healthcare and social welfare entitlements (Figure 5).

**Figure 5:**
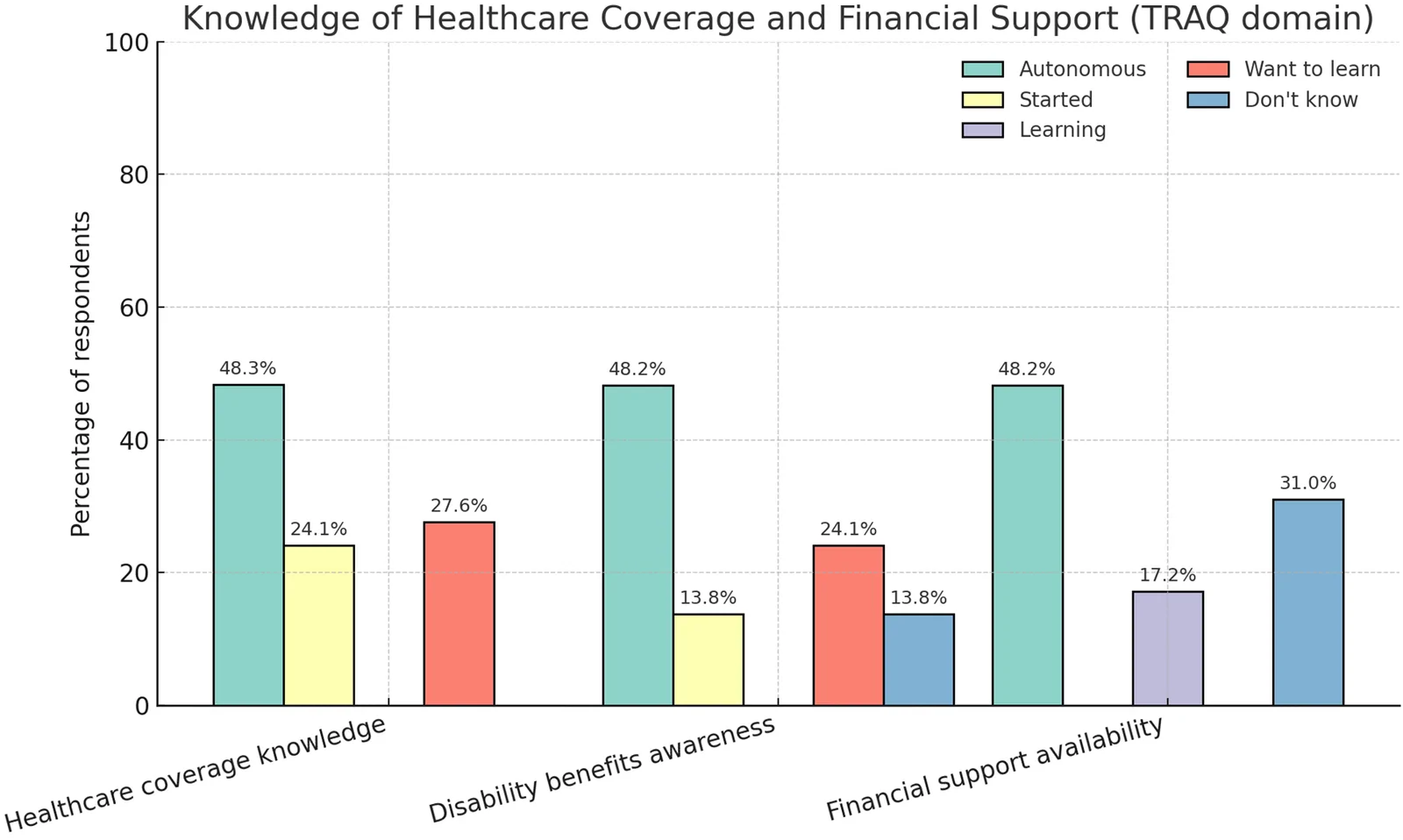
Distribution of responses in the “knowledge of healthcare coverage and financial support” domain of the transition-readiness questionnaire. Bars represent the percentage of participants selecting each response category for the three items assessing knowledge of healthcare coverage, disability benefits, and available financial support.

### 3.3 Structured transition program

#### 3.3.1 Overall transition readiness

Across all questionnaire items, responses revealed a generally high level of transition readiness among participants. Overall, 74.4% of responses corresponded to Level 1 (“I know this”), whereas only 3.8% corresponded to Level 3 (“I need someone for this”). The overall mean score was 1.3 on a 1–3 scale, indicating a high degree of perceived autonomy and competence in managing health and post-transplant care (Figure 6).

**Figure 6:**
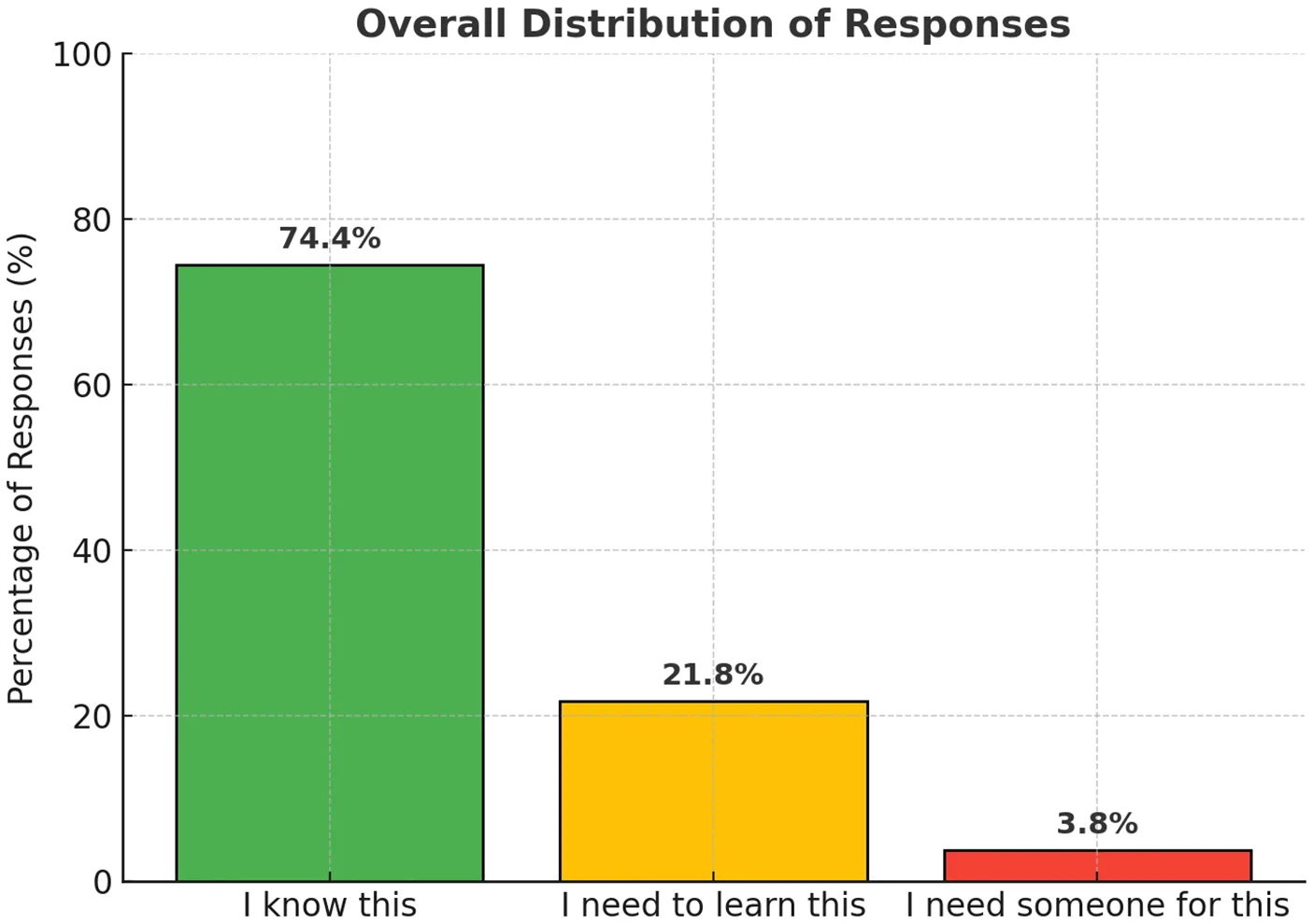
Overall distribution of responses to the transition-readiness questionnaire. Bars represent the overall percentage of responses in each category.

When responses were analyzed according to specific domains, the highest readiness levels were observed in Emotional Management, Lifestyle, and Communication with Healthcare Providers. In contrast, the Medical Care Use domain displayed comparatively lower levels of autonomy and represented the area in which participants most frequently reported the need for further learning and support. The My Health domain showed intermediate results, suggesting that most adolescents had a good understanding of their disease and transplant condition, although some knowledge gaps remained.

Overall, these findings indicated that adolescents nearing transition to adult care exhibit strong readiness in emotional regulation, communication, and daily health-related behaviors, while practical aspects of medical care management remain areas requiring additional support (Figure 7).

**Figure 7:**
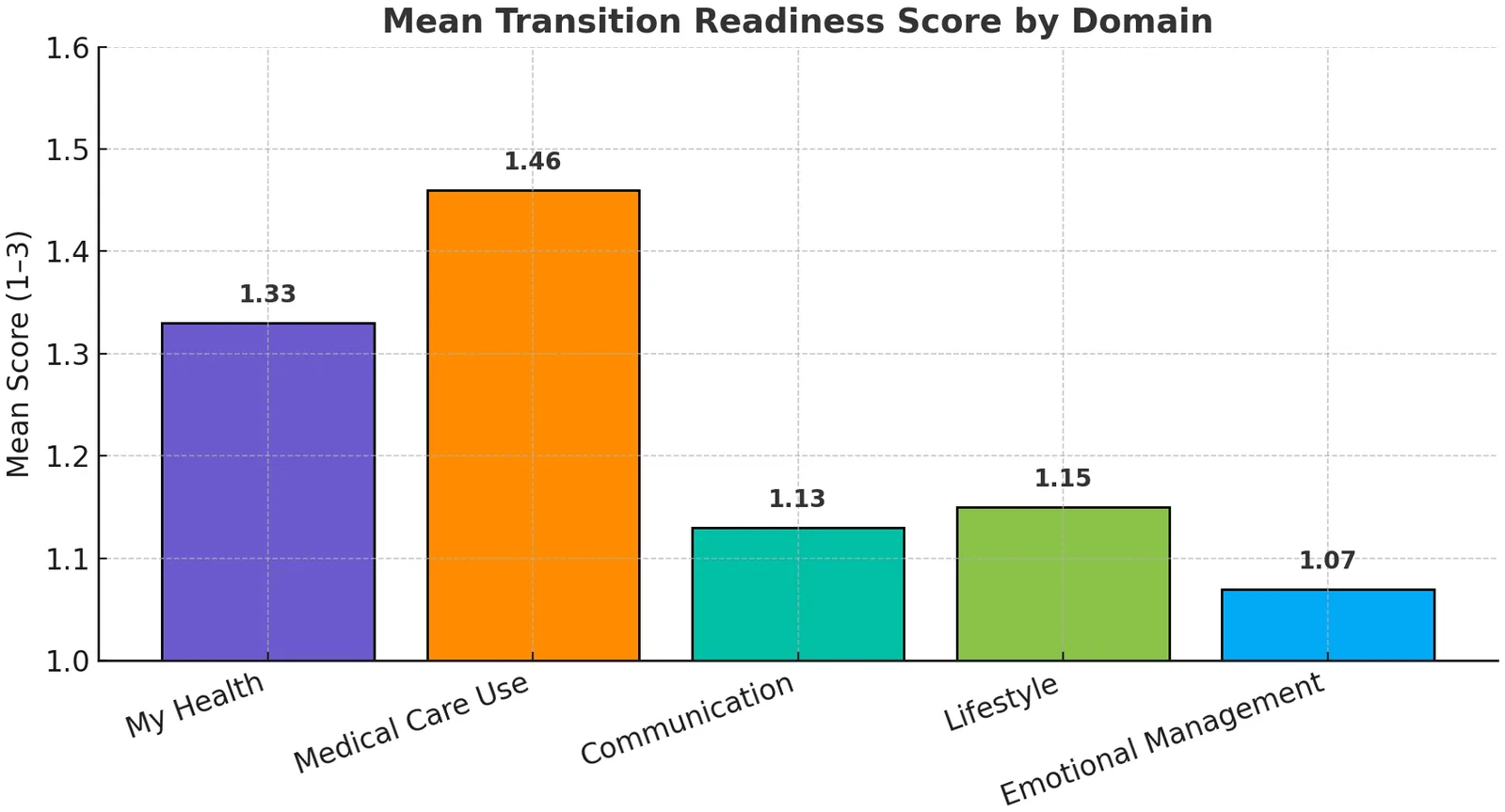
Transition readiness by domain among adolescent kidney transplant recipients*.* Bars represent the mean transition-readiness score reported by adolescents for each domain.

#### 3.3.2 Multidimensional assessment of transition readiness

When comparing responses from adolescents and parents, a largely consistent pattern emerged across the two questionnaires. Parents generally perceived their children as slightly less autonomous than adolescents perceived themselves, particularly in the domains of Medical Care Use and Emotional Management. Overall, adolescents considered themselves ready for transition, whereas parents tended to adopt a more cautious assessment of their level of independence (Figure 8).

**Figure 8:**
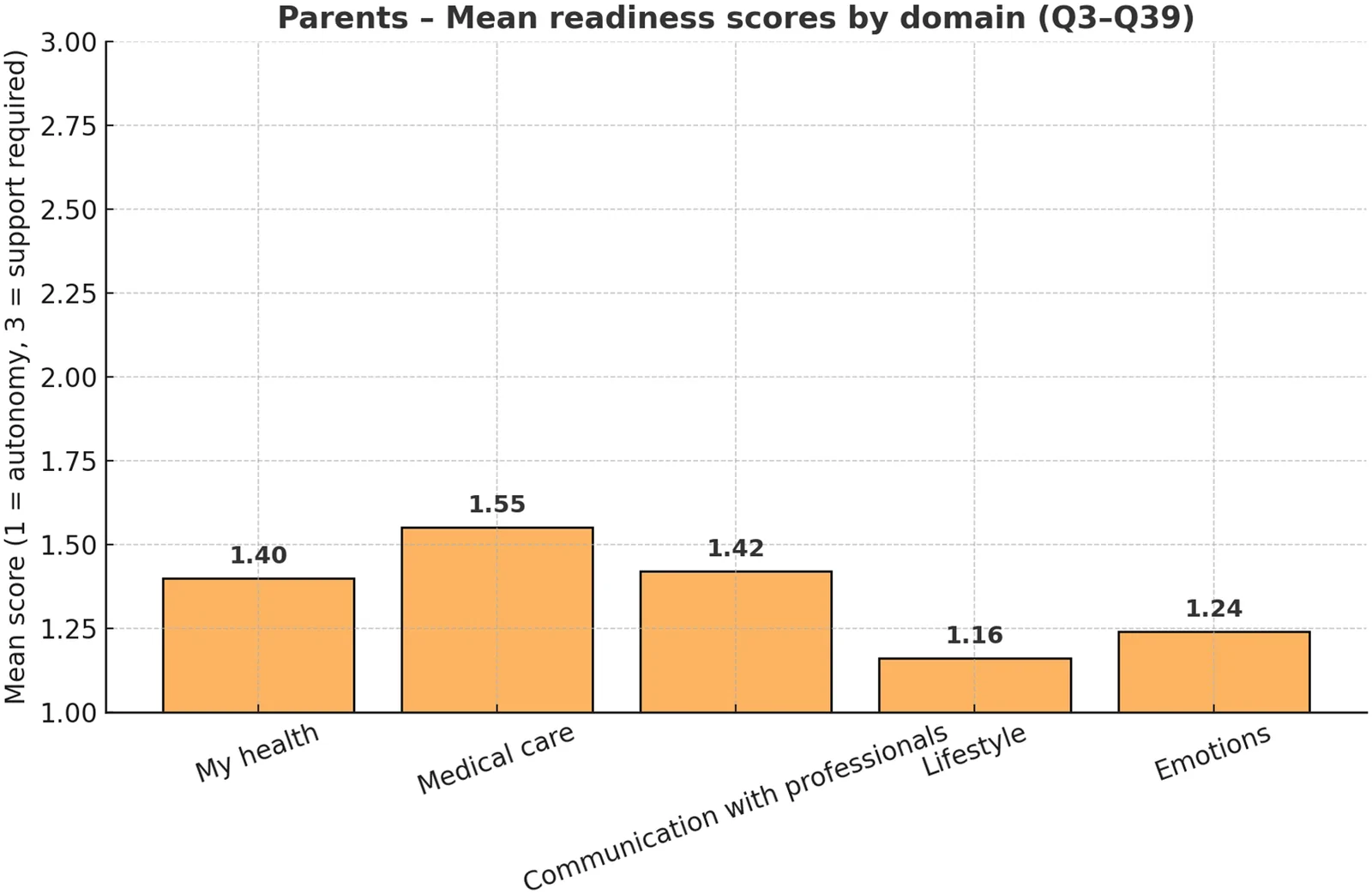
Parents’ mean readiness scores by domain. Bars represent the mean transition-readiness score reported by parents for each domain.

Clinician-reported readiness scores were available for seven patients who had transferred to adult nephrology care. Pediatric nephrologists classified readiness as high in five patients (71%) and intermediate in two patients (29%). According to adult nephrologists, readiness for transition was classified as high in six patients (86%) and intermediate in one patient (14%). No patients were classified as having low readiness.

Overall, pediatric nephrologists tended to assign slightly higher scores, indicating a more cautious assessment of readiness compared with adult nephrologists.

In a subgroup of three patients who had already completed the transition to adult care, transition readiness was assessed from four perspectives: adolescents, parents, pediatric nephrologists, and adult nephrologists. To allow a descriptive comparison across respondents, readiness scores were expressed on a common 1–3 scale, with lower scores indicating greater transition readiness. Overall, the four perspectives showed consistently high levels of readiness, although some variability was observed between patient-, caregiver-, and clinician-reported assessments (Figure 9). Given the small sample size, these findings should be considered exploratory and descriptive.

**Figure 9:**
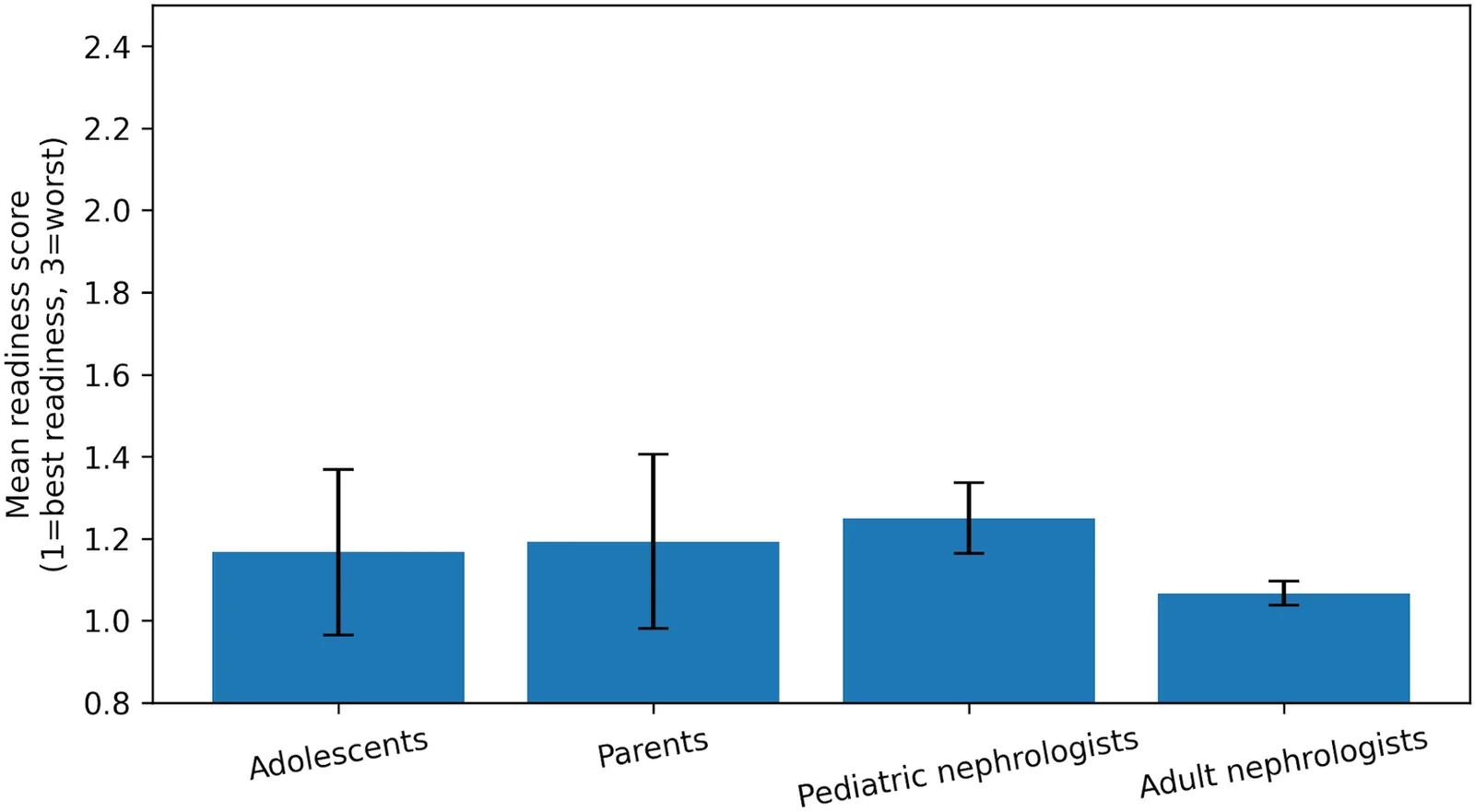
Multiperspective assessment of transition readiness. Comparison of mean transition readiness scores among adolescents, parents, pediatric nephrologists, and adult nephrologists.

#### 3.3.3 Post-transition clinical outcomes

Mean follow-up after transfer to adult care was 7.9 months. During follow-up, graft function remained stable in six of the seven patients evaluated. One patient experienced graft loss and returned to dialysis. No episodes of biopsy-proven acute rejection or major infectious complications were recorded. The patient who experienced graft loss had the highest clinician-reported readiness score, corresponding to an intermediate level of readiness according to both pediatric and adult nephrologists, whereas all patients classified as having high readiness maintained stable graft function during follow-up.

## 4 Discussion

The present study describes the implementation and preliminary evaluation of a structured multidisciplinary transition program for adolescent kidney transplant recipients at a tertiary pediatric kidney transplant center. By integrating a preliminary nursing-led assessment with a structured evaluation of transition readiness, our program provides a multidimensional assessment of transition from the perspectives of adolescents, parents, pediatric nephrologists, and adult nephrologists.

The preliminary nursing-led assessment identified three main findings. First, adolescents demonstrated good autonomy in everyday life and generally good adherence to treatment, with 58.6% reporting autonomous medication use and all participants contributing to routine household activities. Second, the greatest difficulties emerged in the practical management of healthcare: only 17.2% of participants were able to independently request a new prescription or schedule medical appointments, highlighting persistent limitations in healthcare navigation and organizational skills. Third, although communication with healthcare professionals was excellent, with 96.6% of adolescents reporting that they could discuss their health directly with physicians or nurses, knowledge of healthcare entitlements and social support remained heterogeneous, indicating that important educational needs persist beyond disease management. These findings are consistent with previous literature. A qualitative study exploring the transition process after pediatric kidney transplantation identified limited patient autonomy, progressive assumption of responsibility, and difficulties in navigating the healthcare system as major barriers to a successful transition (3). Likewise, recent evidence emphasized that successful transition should be regarded as a gradual developmental process in which adolescents progressively acquire the knowledge, skills, and autonomy required to manage their own healthcare (4, 10). Taken together, these observations suggest that transition programs should specifically promote healthcare navigation, organizational skills, and progressive patient autonomy through targeted educational interventions.

The preliminary evaluation of our structured transition program showed a generally high level of transition readiness among adolescent kidney transplant recipients, with 74.4% of responses indicating complete autonomy (score 1, “I know this”) and an overall mean score of 1.3 on a 1–3 scale. Readiness was highest in the domains of Emotional Management, Lifestyle, and Communication with Healthcare Providers, whereas Medical Care Use remained the least developed domain, with persistent difficulties in medication organization and healthcare navigation. Notably, these findings were consistent with those emerging from the preliminary nursing-led assessment, indicating that practical healthcare management remains a persistent area of vulnerability despite otherwise good levels of self-management. Although few studies have reported detailed domain-specific transition readiness outcomes in pediatric kidney transplant recipients using structured assessment tools, previous studies have shown that transition readiness is closely associated with the progressive assumption of healthcare responsibility during adolescence and with the acquisition of executive skills required for independent disease management, including planning, organization, and self-management abilities (8, 11). Our findings extend these observations by showing that, even in adolescents with an overall high level of transition readiness, practical healthcare management remains the area requiring the greatest educational support. Overall, our findings support the concept that transition readiness is a multidimensional construct rather than a single competency. Adolescents who appear well prepared in terms of disease knowledge, communication, emotional adjustment, and everyday self-management may still require targeted educational interventions to independently organize medical care and effectively navigate adult healthcare services. Structured readiness assessments may therefore help identify specific residual areas of vulnerability and allow transition programs to tailor educational interventions according to individual patient needs.

An additional aspect of our study was the comparison between adolescents’ self-assessments and parents’ perceptions of transition readiness. Overall, the two questionnaires showed a largely consistent pattern, although parents generally perceived their children as slightly less autonomous than adolescents perceived themselves, particularly in the domains of Medical Care Use and Emotional Management. These findings suggest that, despite broad agreement regarding the overall level of readiness, caregivers adopt a more cautious view of adolescents’ ability to independently manage complex healthcare tasks. A previous qualitative study similarly highlighted the gradual transfer of responsibility from parents to adolescents as one of the most challenging aspects of the transition process (12). Parents often experience difficulties relinquishing their central role in disease management, even when adolescents demonstrate increasing autonomy, reflecting the emotional adaptation required not only from patients but also from families (12). Our findings reinforce these observations and support the importance of actively involving parents throughout transition programs, helping them progressively move from direct disease management toward a supportive role that fosters adolescent autonomy. Furthermore, these findings highlight the potential role of healthcare professionals, particularly nurses and psychologists, in mediating expectations, facilitating the gradual handover of responsibilities, and supporting both adolescents and their families through the emotional challenges of the transition process.

A distinctive feature of our study was the comparison between pediatric and adult nephrologists’ assessments of transition readiness. To our knowledge, this is the first study in pediatric kidney transplantation to directly compare the perspectives of pediatric and adult nephrologists using a structured transition readiness assessment. Overall, both specialists considered the adolescents to be well prepared for transfer to adult care, although pediatric nephrologists tended to provide slightly more cautious evaluations than their adult counterparts. This discrepancy may reflect the different timing and perspectives of the two assessments. Pediatric nephrologists evaluated transition readiness at the last pediatric visit, whereas adult nephrologists completed their assessment after a mean follow-up of 7.9 months in adult care, when adolescents had already experienced the transfer process. Furthermore, the pediatric team established longer and closer relationships with the patients during childhood and adolescence, which may provide a more detailed understanding of potential vulnerabilities in treatment adherence or disease self-management. A previous qualitative study in adolescent liver transplant recipients similarly explored the perspectives of pediatric and adult healthcare professionals during transition, showing that the two groups may have different priorities and concerns (13). Pediatric professionals tended to express greater concern about the ability of more vulnerable adolescents to cope after transfer, whereas adult professionals placed greater emphasis on encouraging patient autonomy and progressively reducing parental involvement (13). Our findings extend these observations by providing quantitative evidence in pediatric kidney transplantation and suggest that integrating the complementary perspectives of pediatric and adult nephrologists may enhance the design of multidisciplinary transition programs.

In a subgroup of three transferred adolescents for whom questionnaires were available from multiple informants, we explored transition readiness across four perspectives (adolescent, parent, pediatric clinician, adult clinician). Although based on a very small sample, this exploratory analysis further illustrated the potential value of integrating multiple perspectives when evaluating transition readiness. Incorporating parallel assessments from adolescents, caregivers, pediatric nephrologists, and adult nephrologists represents a novel multi-informant approach that may help identify discordant perceptions and tailor transition interventions according to the individual needs of adolescents and their families.

In addition to transition readiness, our study also evaluated early clinical outcomes following transfer to adult care. Early post-transition outcomes were generally reassuring. During a mean follow-up of 7.9 months, graft function remained stable in almost all patients, with no episodes of biopsy-proven acute rejection or major infectious complications. Overall, these findings are consistent with previous reports showing that structured transition programs can contribute to maintaining favorable post-transfer outcomes despite the well-recognized vulnerability of the transition period for kidney transplant recipients (1, 14). In particular, Weitz et al. reported fewer episodes of acute rejection and a slower decline in graft function after implementation of a standardized multilevel transition program compared with historical controls (15). Similarly, Harden et al. described lower graft failure rates after the introduction of an integrated pediatric-young adult transition clinic (16). In our cohort, the only patient who experienced graft loss had the highest clinician-reported transition readiness score (indicating lower readiness), whereas all patients classified as having high readiness according to both pediatric and adult nephrologists maintained stable graft function. Although this observation should be interpreted with caution because of the limited sample size and short follow-up, it raises the possibility that clinician-reported readiness assessment may help identify adolescents who require closer monitoring and additional support during the transition process. Larger prospective studies with longer follow-up are needed both to validate the potential prognostic value of clinician-reported transition readiness assessment and to determine whether structured multidisciplinary transition programs improve long-term graft outcomes in pediatric kidney transplant recipients.

In our program, transition readiness was assessed in adolescents with a mean age of 18.1 years. Although overall readiness was generally high, several competencies requiring greater autonomy, notably healthcare navigation, independent management of medical care, and communication with healthcare professionals, had not yet been fully achieved by all participants. These findings suggest that the acquisition of some transition-related competencies is a gradual process and that interventions aimed at fostering them should begin earlier during adolescence, rather than shortly before transfer to adult care. There is no universal agreement on the optimal age at which transition planning should begin, and recommendations vary according to the underlying disease and healthcare setting. The American Academy of Pediatrics (AAP) recommends introducing transition planning between 12 and 14 years of age and completing transfer by 18–21 years, while the NICE guidelines advocate initiating preparation around 13–14 years (17, 18). International and European recommendations, including the ISN–IPNA consensus statement and the recent ERA–ESPN Working Groups statement, place greater emphasis on an individualized, developmentally appropriate approach rather than defining a rigid chronological age for transition (6, 19). Our experience also suggests that introducing a structured multidisciplinary transition program at approximately 14–15 years of age may provide sufficient time for the gradual acquisition of the knowledge, self-management skills, and autonomy required before transfer to adult care, while allowing the timing and intensity of transition support to be tailored to the developmental, psychosocial, and clinical needs of each adolescent.

Our findings have direct implications for clinical practice and have already informed further improvements to our transition program. In particular, the readiness assessment identified specific areas of vulnerability, including medication self-management, healthcare navigation, and knowledge of healthcare and social support services, which prompted the introduction of additional targeted interventions. These include nursing-led educational activities focused on medication organization and administration, a practical pocket guide providing information on healthcare exemptions, available benefits, and relevant administrative procedures, and multidisciplinary group sessions involving psychologists, gynecologists, social workers, nurses, and physicians to address broader psychosocial, reproductive, and social aspects of transition. Future longitudinal assessment will be important to determine whether these targeted interventions improve transition readiness and whether such improvements translate into better treatment adherence, continuity of care, and clinical outcomes after transfer.

To our knowledge, this is the first study to evaluate transition readiness in pediatric kidney transplant recipients using a multidimensional approach that integrates the perspectives of adolescents, parents, pediatric nephrologists, and adult nephrologists: the assessment of transition readiness by adult nephrologists provided a unique post-transfer perspective that complemented the pre-transfer evaluations performed by adolescents, parents, and pediatric nephrologists.

Nevertheless, the study was conducted in a single pediatric transplant center and included a relatively small sample, particularly for the subgroup with assessments available from all four informants. As transition readiness was not systematically assessed at both program entry and transfer, longitudinal changes in readiness could not be evaluated in this preliminary analysis. In addition, the relatively short follow-up period precluded evaluation of the long-term impact of the transition program on graft and patient outcomes. Larger multicenter studies with longer follow-up are needed to confirm these findings.

## 5 Conclusions

In conclusion, transition from pediatric to adult care should be considered a gradual developmental process rather than a single transfer event. Our findings suggest that a structured multidisciplinary transition program integrating the perspectives of adolescents, parents, pediatric nephrologists, and adult nephrologists may provide a comprehensive assessment of transition readiness and may help identify areas requiring additional support. Although overall readiness was high, deficits in some self-management skills support the introduction of structured transition interventions early in adolescence to promote autonomy before transfer to adult care. Larger studies are needed to evaluate the long-term impact of structured transition programs on clinical outcomes.

## Data Availability

The original contributions presented in the study are included in the article/Supplementary Material, further inquiries can be directed to the corresponding author.
