# Supplementary material for: Optimizing the transition of care in pediatric kidney transplant recipients at a tertiary center: strategies to enhance autonomy and treatment adherence
Source: Front Pediatr. 2026 Oct 30;14:1971206. doi: 10.3389/fped.2026.1971206 (PMC13759776; doi:10.3389/fped.2026.1971206)
Supplement: Supplementary file 1 [file Table1.docx]

**Table 1. Structure and components of the transition program**

| **Phase** | **Main activities** | **Professionals involved** | **Tools/resources** |
| --- | --- | --- | --- |
| Pre-transition | Introduction to transition pathway; informed consent; readiness assessment; individualized education and empowerment | Pediatric nephrologist, nursing staff ± | Transition-readiness questionnaire |
| Transition | Preparation for transfer and progressive acquisition of autonomy | Pediatric and adult nephrologists; multidisciplinary team (psychologist/social worker/other professionals) as needed | Individualized educational interventions |
| Transfer | At least two joint visits: pediatric setting and adult setting; structured clinical handover | Pediatric and adult nephrologists | Kidney transplant transition checklist/transition report |
| Post-transition | Assessment after transfer | Adult team ± pediatric team | Follow-up assessment |
