# Supplementary material for: Optimizing the transition of care in pediatric kidney transplant recipients at a tertiary center: strategies to enhance autonomy and treatment adherence
Source: Front Pediatr. 2026 Oct 30;14:1971206. doi: 10.3389/fped.2026.1971206 (PMC13759776; doi:10.3389/fped.2026.1971206)
Supplement: Supplementary file 2 [file Table2.docx]

# Supplementary Material – Appendix 2.

# Transition Readiness Assessment for Adolescent Kidney Transplant Recipients

This questionnaire was developed to assess transition readiness among adolescent kidney transplant recipients, focusing on autonomy, self-management, and psychosocial preparedness for transfer to adult care. It includes 39 items divided into five main domains, with a structured multiple-choice response format. Participants were instructed to select the option that best described their current ability or behavior.

## Response scale

| Option | Description |
| --- | --- |
| 1 | Yes, I know how to do this |
| 2 | I need to learn how to do this |
| 3 | I need someone to help me, but I am not sure who |

## Domain 1. My Health

1. 1. I know my diagnosis and understand my disease.
2. 2. I know the name of the medications I take and their purpose.
3. 3. I can describe the side effects of my medications.
4. 4. I take my medications on time without reminders.
5. 5. I know what to do if I miss a dose of my medication.
6. 6. I know how to request a prescription renewal when needed.
7. 7. I can recognize symptoms that require contacting my doctor.
8. 8. I can explain my medical history if asked by a healthcare professional.

## Domain 2. Use of Medical Care

9. 1. I know how to book my medical appointments.
10. 2. I know where to go for my scheduled medical visits.
11. 3. I can plan my transportation for appointments independently.
12. 4. I can report my lab or test results to my medical team.
13. 5. I know how to contact my doctor if I feel unwell.
14. 6. I understand what my health insurance or exemptions cover.
15. 7. I can manage my hospital cards, documents, and health records.

## Domain 3. Communication with Healthcare Professionals

16. 1. I can explain how I feel during medical visits.
17. 2. I can ask questions to my doctor or nurse when I do not understand something.
18. 3. I can describe any changes in my symptoms or side effects to the team.
19. 4. I can express disagreement respectfully if I do not agree with a medical recommendation.
20. 5. I can talk directly with healthcare professionals without relying on my parents.

## Domain 4. Lifestyle and Daily Activities

21. 1. I can prepare simple meals independently.
22. 2. I can manage my personal hygiene and household tasks.
23. 3. I can handle my personal expenses and use money responsibly.
24. 4. I can plan my school or work schedule around medical appointments.
25. 5. I can manage my sleep schedule and physical activity for my health.
26. 6. I understand the importance of avoiding smoking, alcohol, and risky behaviors.
27. 7. I know who to contact if I need social or educational support.

## Domain 5. Emotional Management

28. 1. I can recognize when I feel stressed or anxious.
29. 2. I know strategies to calm myself when I feel upset.
30. 3. I can talk about my emotions with a trusted person.
31. 4. I know who to contact if I feel emotionally unwell.
32. 5. I know where to find psychological support if needed.
33. 6. I feel confident about my future and ability to manage my health.
34. 7. I am aware that the transition to adult care will bring new responsibilities.

Questionnaire responses were collected during outpatient follow-up visits and analyzed using descriptive statistics (means, standard deviations, and frequency distributions) to evaluate overall readiness and identify specific areas requiring further educational or psychological support.
