# Supplementary material for: Optimizing the transition of care in pediatric kidney transplant recipients at a tertiary center: strategies to enhance autonomy and treatment adherence
Source: Front Pediatr. 2026 Oct 30;14:1971206. doi: 10.3389/fped.2026.1971206 (PMC13759776; doi:10.3389/fped.2026.1971206)
Supplement: Supplementary file 3 [file Table3.docx]

# Supplementary Material – Appendix 3. Transition Readiness Questionnaire for Parents of Adolescent Kidney Transplant Recipients

This questionnaire was designed to assess parental perspectives on their adolescent’s readiness for transition from pediatric to adult kidney transplant care. It explores autonomy, self-management, communication, and emotional aspects, from the parents’ point of view. Parents were invited to select the response that best describes their child’s current ability or behavior in each area.

## Response scale

| Option | Description |
| --- | --- |
| 1 | Yes, my child knows how to do this |
| 2 | My child needs to learn how to do this |
| 3 | My child needs someone to help, but is not sure who |

## Domain 1. My Child’s Health

1. 1. My child knows and understands their diagnosis.
2. 2. My child knows the name and purpose of their medications.
3. 3. My child is able to take medications on time without being reminded.
4. 4. My child knows what to do in case of side effects or missed doses.
5. 5. My child can describe their medical history to healthcare professionals.
6. 6. My child is aware of which symptoms require contacting a doctor.

## Domain 2. Relationship with the Healthcare Team

7. 1. My child can book their own medical appointments.
8. 2. My child can attend medical visits without my presence.
9. 3. My child can contact the medical team if their health changes suddenly.
10. 4. My child knows how to renew prescriptions or medical certificates.
11. 5. My child understands what their healthcare coverage includes (e.g., exemptions).
12. 6. My child keeps a record of visits, tests, and results.

## Domain 3. Communication with Professionals

13. 1. My child can explain how they feel to doctors and nurses.
14. 2. My child can ask questions or seek clarification during visits.
15. 3. My child answers independently when healthcare professionals ask questions.
16. 4. My child can discuss concerns without me prompting them.
17. 5. My child can express disagreement respectfully when they do not agree with a medical recommendation.

## Domain 4. Lifestyle and Daily Activities

18. 1. My child manages daily activities independently (personal hygiene, meals, house tasks).
19. 2. My child can manage their own schedule (school, hobbies, appointments).
20. 3. My child can organize transportation to medical visits.
21. 4. My child knows how to handle money responsibly.
22. 5. My child avoids risky behaviors such as smoking or alcohol use.
23. 6. My child knows who to contact for school or social support if needed.

## Domain 5. Emotional Management

24. 1. My child recognizes when they feel anxious, angry, or sad.
25. 2. My child knows how to calm down when feeling upset.
26. 3. My child can talk about their emotions with a trusted person.
27. 4. My child knows who to contact if they need psychological help.
28. 5. My child seems confident about their ability to manage health in adult care.

Parents completed the questionnaire during outpatient follow-up visits. Responses were analyzed using descriptive statistics (means, standard deviations, and frequencies) to identify discrepancies between parental perceptions and patient self-assessment, and to guide educational interventions.
