# Supplementary material for: Optimizing the transition of care in pediatric kidney transplant recipients at a tertiary center: strategies to enhance autonomy and treatment adherence
Source: Front Pediatr. 2026 Oct 30;14:1971206. doi: 10.3389/fped.2026.1971206 (PMC13759776; doi:10.3389/fped.2026.1971206)
Supplement: Supplementary file 4 [file Table4.docx]

**Supplementary Material – Appendix 4. Transition Readiness Questionnaire Nephrologist Assessment**

Instructions for completion:
This questionnaire is completed by the nephrologist at the end of the clinical visit.
For each item, assign a score using a reverse scale:
1 = Adequate (highest level of readiness)
2 = Partially adequate
3 = Not adequate (lowest level of readiness)

# Section A – Disease Awareness (range 4–12)

Knows the nephrological diagnosis	☐ 1	☐ 2	☐ 3

Understands the chronic nature of the disease	☐ 1	☐ 2	☐ 3

Is aware of relevant clinical history	☐ 1	☐ 2	☐ 3

Recognizes major warning signs	☐ 1	☐ 2	☐ 3

Section total: ______

# Section B – Therapy and Adherence (range 5–15)

Knows current medications	☐ 1	☐ 2	☐ 3

Knows the indication for each medication	☐ 1	☐ 2	☐ 3

Knows dosage and administration schedule	☐ 1	☐ 2	☐ 3

Manages therapy independently	☐ 1	☐ 2	☐ 3

Demonstrates adequate treatment adherence	☐ 1	☐ 2	☐ 3

Section total: ______

# Section C – Follow-up and Monitoring (range 4–12)

Understands the need for regular follow-up	☐ 1	☐ 2	☐ 3

Knows which tests are required	☐ 1	☐ 2	☐ 3

Knows where to undergo tests and visits	☐ 1	☐ 2	☐ 3

Is able to report clinically relevant results	☐ 1	☐ 2	☐ 3

Section total: ______

# Section D – Use of Healthcare Services (range 5–15)

Knows how to contact the nephrology center	☐ 1	☐ 2	☐ 3

Knows how to access urgent care services	☐ 1	☐ 2	☐ 3

Knows their general practitioner (GP)	☐ 1	☐ 2	☐ 3

Is aware of healthcare exemptions/certifications	☐ 1	☐ 2	☐ 3

Has updated medical documentation available	☐ 1	☐ 2	☐ 3

Section total: ______

# Section E – Transition to Adult Care (range 3–9)

Understands the shift in healthcare responsibility	☐ 1	☐ 2	☐ 3

Understands the concept of informed consent	☐ 1	☐ 2	☐ 3

Is informed about transfer to adult care services	☐ 1	☐ 2	☐ 3

Section total: ______

# Total Score

Total range: 21 (best readiness) – 63 (lowest readiness)

Suggested categories:
21–30 → High transition readiness
31–45 → Intermediate readiness
>45 → Low transition readiness
